# Functional analysis of a novel variant in the *COL5A1* gene in a Polish patient with the classical type of Ehlers–Danlos syndrome

**DOI:** 10.3389/fgene.2025.1689587

**Published:** 2025-11-06

**Authors:** Anna Junkiert-Czarnecka, Karolina Maciak, Magdalena M. Kacprzak, Agnieszka Łobodzińska, Agnieszka Sobczyńska-Tomaszewska, Aneta Jurkiewicz, Monika Gora, Beata Burzynska, Maria Pilarska-Deltow, Olga Haus

**Affiliations:** 1 Department of Clinical Genetics Collegium Medicum in Bydgoszcz, Nicolaus Copernicus University in Torun, Bydgoszcz, Poland; 2 Institute of Biochemistry and Biophysics, Polish Academy of Sciences, Warsaw, Poland; 3 Centrum Medyczne MEDGEN, Warsaw, Poland

**Keywords:** Ehlers–Danlos syndrome, next-generation sequencing, functional analysis, minigene, collagen

## Abstract

Ehlers–Danlos syndrome (EDS) is a clinically and genetically diverse group of inherited connective tissue disorders. According to the 2017 International Classification, 13 EDS subtypes are associated with pathogenic variants in 19 genes, most of which are involved in collagen synthesis or structure. The most common forms include classical (cEDS) and hypermobile (hEDS) types. Classical EDS is primarily caused by pathogenic variants in the *COL5A1* and *COL5A2* genes, which encode type V collagen, and less frequently by the c.934C>T variant in COL1A1. This study investigated the molecular basis of cEDS in a 9-year-old girl presenting clinical features consistent with this subtype. Whole-exome sequencing (WES) identified a novel variant in COL5A1: c.2089-7_2089dupGTACACAG. Functional analysis showed that this duplication causes a shift in the exon start site, resulting in a premature stop codon and a predicted truncated protein lacking approximately 1,000 amino acids. Family studies confirmed that the variant occurred *de novo*. This pathogenic variant, located in the triple helical domain of type V collagen, likely disrupts the final structure and function of the protein. The two-step diagnostic strategy combining molecular and functional testing enabled a rapid and definitive diagnosis for the patient.

## Introduction

Ehlers–Danlos syndrome (EDS) is a heterogeneous group of heritable connective tissue disorders. The 2017 International Classification of EDS recognizes 13 subtypes caused by pathogenic variants in 19 genes, encoding different types of collagen or proteins involved in collagen metabolism. The most abundant types of EDS are classical (cEDS) and hypermobile (hEDS) (Malfait et al., 2017). The leading causes of classical Ehlers–Danlos syndrome are pathogenic variants in *COL5A1* and *COL5A2*, which encode type V collagen, and the c.934C>T variant in the *COL1A1* gene (Symoens et al., 2012). According to the classification, the following criteria must be met to establish a clinical diagnosis:

### Major criteria

- Significant skin hyperextensibility and atrophic scarring
- Generalized joint hypermobility

### Minor criteria

- Easy bruising
- Soft, doughy skin
- Skin fragility (or traumatic splitting)
- Molluscoid pseudotumors
- Subcutaneous spheroids
- Hernia (or history thereof)
- Epicanthal folds
- Complications of joint hypermobility (e.g., sprains, dislocation/subluxation, pain, and pes planus)
- Family history of a first-degree relative who meets clinical criteria.

Minimal criteria suggestive of a diagnosis of classical EDS: major criteria include skin hyperextensibility and atrophic scarring, along with either the major criterion generalized joint hypermobility or three of the nine minor criteria (Bowen et al., 2017).

The clinical features included in the EDS classification represent only a portion of all the disabilities recognized in EDS patients. In addition to major and minor phenotypic criteria, patients’ clinical presentations include dysfunctions of the gastrointestinal, cardiovascular, immune, neural, and other systems. The clinical symptoms may differ among patients and within the same family and may occur at different ages or with various intensities. Because of phenotypic heterogeneity and clinical overlap among EDS types and between EDS and other connective tissue disorders, molecular testing with next-generation sequencing (NGS) with a multigene panel of connective tissue-associated genes or whole-exome sequencing (WES) seems to be an adequate method.

The present study evaluated the molecular background of a 9-year-old girl with cEDS symptoms.

## Materials and methods

### Patient

In this investigation, we examined a 9-year-old girl born pre-term (36 hbd; length: 49 cm; weight: 2,390 g) who presented with hypermobile joints (8/9 in Beighton score), with no dislocations, delayed motor development (sitting at 9 months and walking at 18 months), and soft, doughy, hyperextensible skin with a tendency to injury and delayed wound healing, but no atrophic scarring. The patient also presented malocclusion, epicanthal folds, blue sclera, hernias, pes planus, abdominal pain (without a determined cause; the diagnosis for celiac disease was negative), and a Baker’s cyst. Moreover, the patient’s height and weight were below the 10th percentile grids. The patient’s mother indicated a high tendency for recurrent infections such as flu type A, type B, COVID-19, otitis, laryngitis, and bronchitis. Before genetic testing, the patient was evaluated by a cardiologist, neurologist, ophthalmologist, and gastroenterologist–all scores were standard. Family history (parents and younger brother) was negative for EDS symptoms.

### DNA sequencing and *in silico* prediction

WES was performed. The patient’s genomic DNA was extracted from the whole-blood sample, and the sequencing library was prepared according to the Twist Human Core Exome Plus Kit (Twist Bioscience) protocol. The enriched DNA libraries were sequenced using the Illumina NovaSeq 6000 Instrument (Illumina, Inc., San Diego, CA, United States). All procedures for exome sequencing were conducted by CeGaT (Germany). Raw sequencing reads were mapped to the reference genome using BWA-MEM2 v2.2.1 (Li and Durbin, 2010). Duplicates were removed using Picard 2.18.2-SNAPSHOT software (Broad Institute, Cambridge, MA, United States); variants were called using HaplotypeCaller: gatk-4.2.6.1, annotated with VEP 105, and named using SAMtools software (SourceForge, San Diego, CA, United States). The following *in silico* prediction software programs were used to assist with the interpretation of the pathogenicity of the detected variant: Alamut Visual Plus 1.7.2 (Interactive software, SOPHiA GENETICS, Switzerland), https://franklin.genoox.com, https://genebe.net/, and others. The presence of the variant in control populations was checked in dbSNP (Sherry et al., 1999) and gnomAD (Broad Institute).

### Analysis of the effect of the identified variant on splicing

#### Minigene constructs

To study the effect of the detected variant, we constructed a wild-type and a mutated hybrid minigene using the vector pSpliceExpress (Addgene: #32485, a kind gift from Stefan Stamm) (Kishore et al., 2008). The genomic DNA region containing *COL5A1* exon 22, along with the flanking 5′ and 3′ intronic sequences (>150 bp), was PCR-amplified from the control and patient DNA using a proofreading DNA polymerase. The forward primer (Fwd_COL5A1-pSE-XhoI; 5′-ATC​ATC​CTC​GAG​CAG​CCC​CGT​GAC​CTG​GTT​TT-3′) contained the XhoI site (bolded), and the reverse primer (Rev_ COL5A1-pSE-NotI; 5′-ATT​ATC​GCG​GCC​GCC​CAG​ACA​TCC​GAC​CAG​GAA​G-3′) contained the NotI site (bolded). NotI and XhoI restriction sites were used to insert the PCR products and construct the minigene vectors. Sanger sequencing of the whole exon and flanking intronic sequences verified the final constructs.

#### Cell culture and transfection

Flp-In-293 cells (2.5 × 10^5^) were transfected with 1 µg of plasmid DNA carrying either the reference allele or variant allele of interest using TransIT®-2020 Transfection Reagent (Mirus Bio LLC, Madison, WI, United States). Cells were harvested 24 h after transfection. Total RNA was extracted using the total RNA Mini Plus (A&A Biotechnology, Gdynia, Poland), and the RNA samples were quantified using spectrophotometry. cDNA synthesis was performed using the TranScriba noGenome Kit (A&A Biotechnology).

#### Transcript amplification

cDNA was PCR-amplified using previously reported PCR primers specific for the rat insulin 2 exons (Ins2-F: 5′-AGG​TCT​GAA​GGT​CAC​GGG​CC-3′ and Ins2-R: 5′-CCT​GCT​CAT​CCT​CTG​GGA​GC-3) (Tang et al., 2020). Products were visualized using agarose gel electrophoresis. In addition, DNA fragments generated using PCR amplification were sequenced to confirm splicing products.

### Variant interpretation

The pathogenicity of the identified variant was classified using the recommendations of American College of Medical Genetics and Genomics and the Association for Molecular Pathology (ACMG–AMP) (Richards et al., 2015).

## Results

### Genetic analysis and *in silico* prediction

**FIGURE 1:**
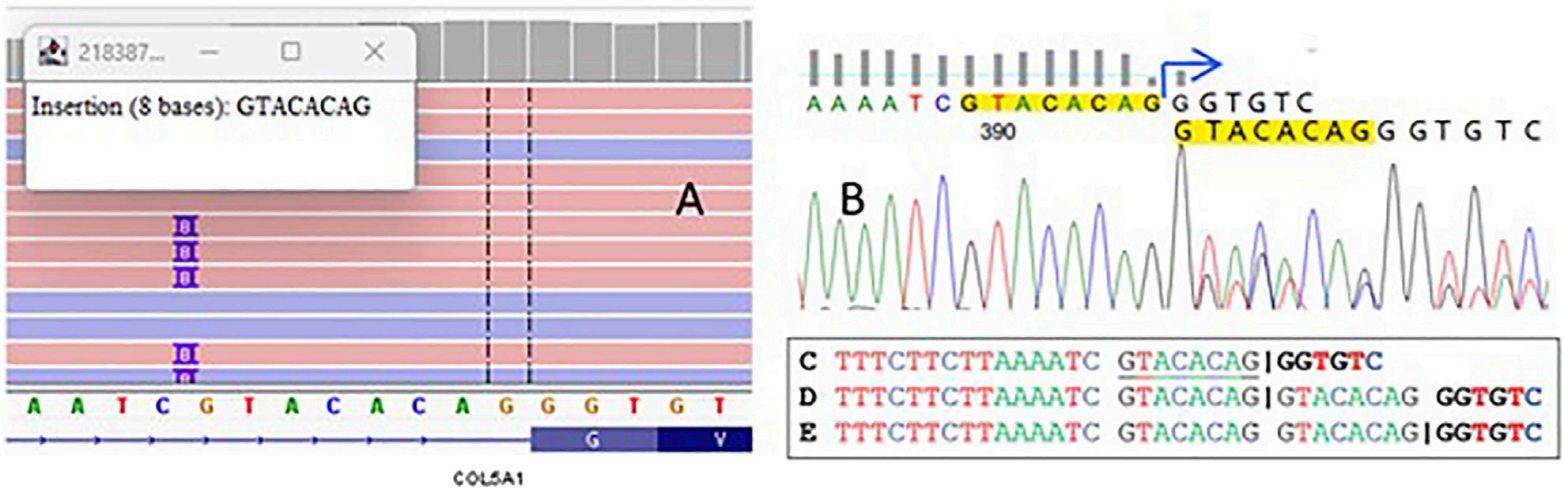
A visualization of sequencing reads that demonstrate an insertion of eight nucleotides in the splicing region of the *COL5A1* gene **(A)**. The electropherogram plot from Sanger sequencing confirms this variant **(B)**. The splice site in the wild-type sequence is shown by the “|” symbol C, along with the potential impact of the duplication on the positioning of splice sites in the mutant allele D,E.

NGS examination revealed the variant chr9-134766445-C-CGTACACAG (GRCh38) c.2089-7_2089dupGTACACAG in the *COL5A1* gene (Figure 1A) located in the polypyrimidine tract upstream from exon 22 (according to NM_000093.5). Sanger sequencing was used to confirm the presence of the variant in the patient (Figure 1B).

Bioinformatic tools assessed the duplication as deleterious, predicting a frameshift that introduces a premature termination codon after 110 nucleotides (p.Gly697Valfs*110). To determine the real impact of the variant, we performed functional studies to validate the predictions from Alamut and SpliceAI, which indicated loss of the native acceptor site (delta 0.95) and gain of a novel acceptor site (delta 0.99), resulting in an 8 bp extension of exon 22—a crucial finding for establishing the patient’s final diagnosis.

The variant identified in the present study was not reported in LOVD (lovd.nl), gnomAD (gnomad.broadinstitute.org), and dbSNP (ncbi.nlm.nih.gov/snp) databases. It has, however, been submitted to the ClinVar database by one of the co-authors of this study (https://www.ncbi.nlm.nih.gov/clinvar/variation/3338117/).

According to the ACMG–AMP guidelines, the variant was classified as likely pathogenic, meeting the following criteria: PM2—absent from gnomAD genomes with good coverage and PVS1—a null (frameshift) variant in the *COL5A1* gene, where loss of function is a known disease mechanism. However, the detected duplication can be interpreted in two distinct ways. According to bioinformatic predictors, it may impact the coding sequence (Figure 1), potentially resulting in a frameshift that alters the protein. Alternatively, the duplication might only affect a fragment of an intron, preserving the original splice site while modifying only the polypyrimidine tract (Figure 1). To determine the real impact of the variant, we decided to conduct functional studies on the variant and validate the accuracy of Alamut and other SpliceAI predictions, which was essential for establishing the diagnosis of patient.

### Patient’s investigation

**FIGURE 2:**
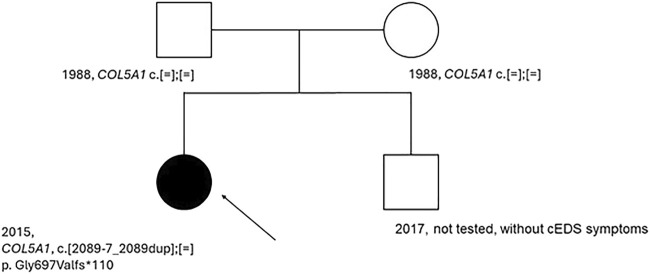
Patient’s pedigree. An arrow indicated the proband. Dark circle, symptomatic member of the family; light circle and square, asymptomatic member of the family; c.[=];[=], absence of the variant, c.[2089-7_2089 dup];[=], presence of the variant on one allele; *COL5A1*, name of the tested gene.

Testing for the variant *COL5A1* c.2089-7_2089dup was conducted on the patient and her parents. The parents were asymptomatic of cEDS, possibly not carriers of the *COL5A1* alteration. The test was not performed on the younger brother, who did not show cEDS symptoms (Figure 2).

Negative results for the presence of variants in the patient’s parents indicate that this is a *de novo* variant. In the next step, functional studies were conducted to confirm its pathogenicity and role in the pathogenesis of cEDS.

### *COL5A1* minigene

**FIGURE 3:**
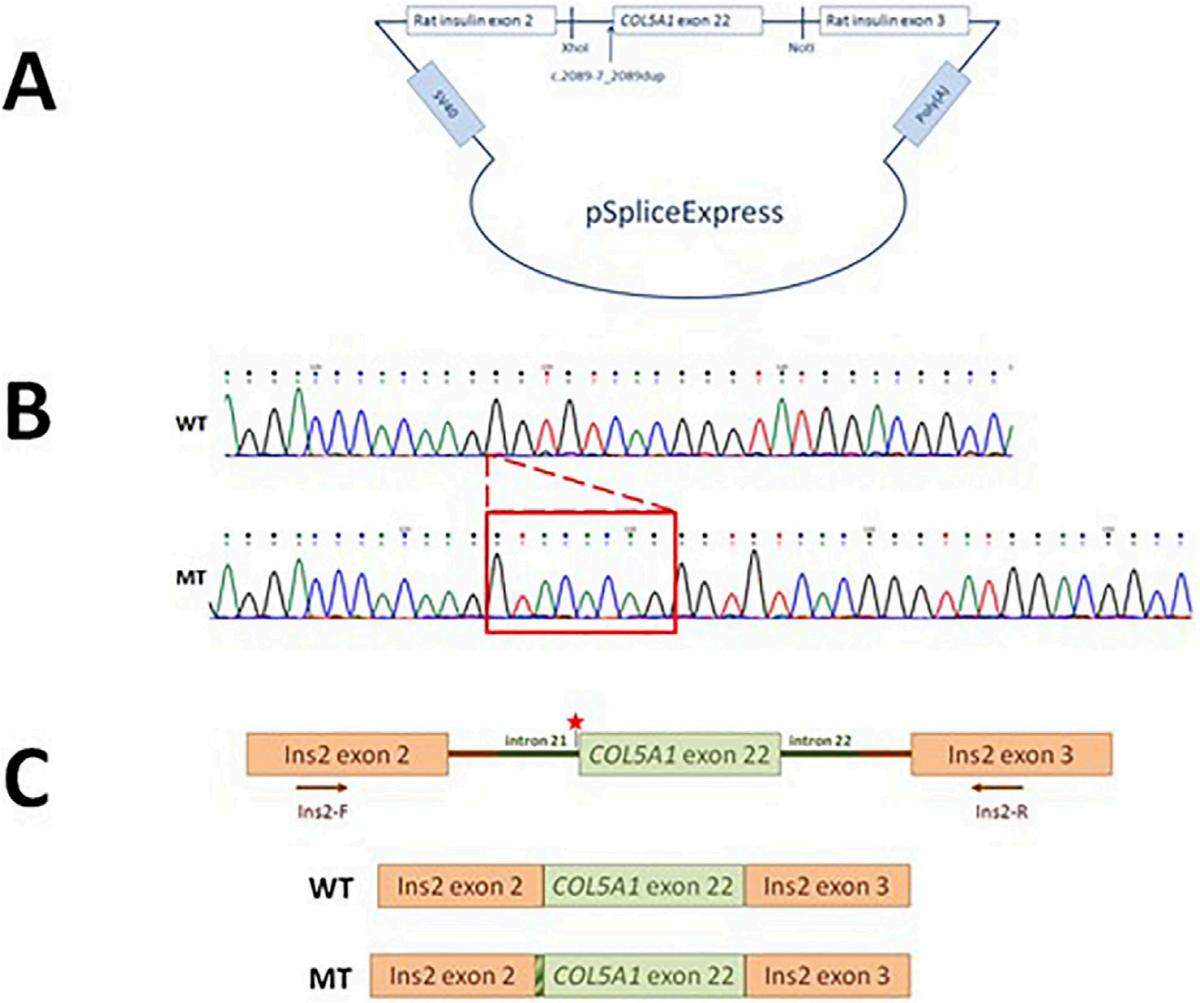
Characteristics of the *COL5A1* variant. **(A)** The pSpliceExpress vector containing a fragment of the *COL5A1* gene with the c.2089-7_2089 dup variant with flanking introns was cloned between two rat insulin exons by NotI- and XhoI-compatible overhangs. **(B)** Sanger sequencing of the WT and mutated transcript. **(C)** Schematic presentation of the WT and mutated minigene constructs.

A schematic representation of the *COL5A1* minigene construct used to analyze the effect of the c.2089-7_2089 dup variant on pre-mRNA splicing is shown in Figure 3A–C. The PCR products of cDNA synthesized from total RNA extracted from Flp-In-293 cells transfected with the *COL5A1* minigene constructs produced one splicing product of approximately 240 bp for the WT and mutant constructs. Sequencing confirmed that an 8-nucleotide duplication (GTACACAG) upstream of exon 22 leads to the exon starting earlier and, in consequence, introduces a termination codon into the reading frame and is predicted to encode a truncated protein by 1,000 amino acids (Figure 3).

## Discussion

In approximately 50% of patients with classical EDS, a variant in two of the three genes for type V collagen, *COL5A1* and *COL5A2*, occurs. The *COL5A1* gene is located at 9q34.2–q34.3 and comprises 66 exons over >150 kb of gDNA, encoding the α1 chain of type V collagen. Approximately one-third of individuals with classical EDS have nonsense or frameshift variants, leading to the non-functional *COL5A1* allele (Love and Palmer, 2023). The most common substitutions are variants consisting of the substitution of glycines in the triple helix domain of the glycine, which have harmful effects by producing an abnormal α-chain (Monroe et al., 2015).

*De novo* variants are uncommon, but some cases have been documented in the literature. In particular, molecular characterization of 40 patients with classical Ehlers–Danlos syndrome resulted in the identification of 18 *COL5A1* variants, including two *de novo* alterations that produced in-frame skipping of the affected exon (Ritelli et al., 2013).

According to the ClinVar database (https://www.ncbi.nlm.nih.gov/clinvar), 249 variants have been classified as pathogenic and 102 as likely pathogenic. In our patient, we detected a novel intronic variant in a heterozygous state. ACMG–AMP recommends the laboratory verification of splicing variants through functional RNA or protein analysis. Technical approaches include direct RNA and complementary DNA derivative analysis, *in vitro* minigene constructs, and splicing assays (Richards et al., 2015).

Following these guidelines, we performed functional analyses of the detected variant utilizing the minigene approach. For the *de novo* variant, where there is no family history and the possibility of conducting familial segregation studies, only functional tests allow for a definitive determination of the role of the detected variant. Type V collagen has a regulatory function in fibrillogenesis. Therefore, pathogenic variants in genes *COL5A1* and *COL5A2* coding for V-type collagen play a significant role in the pathogenesis of classical EDS. The analyzed variant, *COL5A1* c.2089-7_2089dup (p.Gly697Valfs*110), is located in the functional triple helix domain of the V-type collagen molecule and impacts its final structure and function (Paladin et al., 2015). We used minigene assays in HEK293T cells to assess the variant’s pathogenicity. Although this system provided an efficient platform for our functional analyses, it is essential to acknowledge its limitations. HEK293T cells lack tissue-specific collagen quality control mechanisms, present altered post-translational modification profiles, and display non-physiological extracellular assembly compared to native collagen-producing cells (Roulet et al., 2010; DiChiara et al., 2016; Schwarz, 2015). Nonetheless, for the specific purpose of evaluating the effect of the detected variant on the splicing process, HEK293 cells represent a well-established and reliable model. They consistently produce correctly processed transcripts and proteins from transfected minigenes, and results from such assays reflect physiologically relevant splicing outcomes. This approach has, therefore, become a standard in evaluating splice variants and reporter constructs, particularly when patient RNA is unavailable or bioinformatic predictions are non-definitive, and it may also be applied as part of routine diagnostic procedures.

According to the ACMG–AMP guidelines, the identified variant initially meets the criteria for classification as likely pathogenic (PVS1 and PM2). However, when considering the *de novo* occurrence (PM6) and the results of the minigene functional assay providing strong experimental evidence (PS3), this variant can be reclassified as pathogenic. Therefore, the functional analysis confirmed the splicing alteration predicted by Alamut and SpliceAI and provided the critical evidence required for final variant classification and establishing the patient’s diagnosis.

Thus, minigene functional assays are essential to confirm the pathogenicity of splice variants when patient RNA or fibroblasts are not available— — which is often the case— — and they also provide a fast, reliable, and increasingly routine tool in the molecular diagnostics of EDS and other connective tissue disorders. In the era of next-generation sequencing, where numerous variants are detected and their significance can be difficult to interpret, a two-step diagnostic approach combining molecular and functional testing allows for a rapid and reliable confirmation of the final diagnosis (Walker et al., 2023; Fraile-Bethencourt et al., 2019; Jaganathan et al., 2019).

## Conclusion

Thus, minigene functional assays are essential to confirm the pathogenicity of splice variants when patient RNA or fibroblasts are not available—which is often the case—and they also provide a fast, reliable, and increasingly routine tool in the molecular diagnostics of EDS and other connective tissue disorders. In the era of NGS, where many variants are detected and their significance can be difficult to interpret, a two-step diagnostic approach combining molecular and functional testing allows for a rapid and reliable confirmation of the final diagnosis.

## Data Availability

The datasets presented in this study can be found in online repositories. The names of the repository/repositories and accession number(s) can be found below: https://www.ncbi.nlm.nih.gov/ www.ncbi.nlm.nih.gov/clinvar/RCV004698462, https://www.ncbi.nlm.nih.gov/clinvar/variation/3338117/.

## Abbreviations

EDS, Ehlers–Danlos syndrome; cEDS, classical Ehlers–Danlos syndrome, hEDS, hypermobile Ehlers–Danlos syndrome; NGS, next-generation sequencing; WES, whole-exome sequencing; ACMG–AMP, American College of Medical Genetics and Genomics and Association for Molecular Pathology.
